# Solid Leaven

**Published:** 1875-10

**Authors:** 


### SOLID LEAVEN.

The use of yeast, or some kindred substance, to render bread light and porous, has existed among all nations. By its employment little air-cells are created in the paste, giving a spongy appearance to the bread. The bulk is thereby increased, and the appearance is improved, to the ordinary eye. If the leavening or raising process be not too long continued, the taste of the product is not impaired, and bread which might otherwise be soggy, being inflated by the carbonic gas of fermentation, is light and soft.

Some people do not like bread which has been permeated and inflated with yeast bubbles; others will not eat any other kind if they can help it. Perhaps a little information upon this yeast topic will be productive of good.

The value of yeast in making spongy bread, rests in the fact that it contains in itself the active fermentive power. If time enough were allowed, the batch of bread would ferment without the aid of any leaven whatever. But good housewives who have tried all methods, declare that bread which ferments slowly and in the natural way, is not as pleasing to the eye, or as grateful to the palate, or as nourishing to the body, in their judgment, as bread which has been forced into active and speedy fermentation by the addition of suitable leaven.

Now if yeast is used, it would seem important that it should be good and pure. Brewer's yeast, which is widely used, has its objections. All fluid yeasts must be uncertain in operation, because of the danger that they wil' pass beyond the initial fermentive stage to the stage of active and absolute decomposition, when they become unwholesome infusions.

From the researches of Mr. W. H. Burton, of the establishment known as the Waterloo Yeast Company, it has been made evident that solid, dry yeast, made of the proper cereal and compressed into cakes, is the most wholesome and trustworthy, as well as the most convenient form which leaven can assume. The reason for the superiority of solid yeast rests in the fact that the succeeding changes which nature has provided for all vegetable substances after the fermentive stage begins, are arrested and held in abeyance by the mode of preparation pursued. There is no diminution of the activity of the principle; it is merely a suspension of the power until the moment comes for its putting forth. That moment arrives whenever moisture is applied and the presence of dough or other suitable substance exists. Just where the work was arrested months before, it is recommenced. With proper watchfulness, there is no danger of destroying the batch of bread by the use of this leaven. The risk of inducing decomposition, and securing a mass of sour and worthless bread, is thus reduced to a minimum.

The grain used by this Company is corn. Choice corn is selected and ground to a powder, and saturated with a sweet ferment made from fresh hops and soft water. This leavened dough is then moulded into cakes, and quickly dried. The meal thus absorbs and protects the fermentive principle; and for a long period no chemical change occurs in the cake thus formed. It is white in color, and it imparts no tinge to the bread into which it enters.

The business done by the Waterloo Yeast Company in the manufacture of the product which we have spoken of, called the "Yankee Dry Hop-Yeast," is very large. The factory is at No. 48 Hudson Street, New York. The price affixed to this yeast is no greater than is demanded by dealers for an inferior article. Almost any good grocer will sell you this yeast, and will tell you that it has a high reputation among the best classes of those who use leavened bread as an article of diet.

